# Therapeutic window of globular adiponectin against cerebral ischemia in diabetic mice: the role of dynamic alteration of adiponectin/adiponectin receptor expression

**DOI:** 10.1038/srep17310

**Published:** 2015-11-27

**Authors:** Wenying Song, Fan Guo, Haixing Zhong, Lixin Liu, Rui Yang, Qiang Wang, Lize Xiong

**Affiliations:** 1 Department of Anesthesiology, Xijing Hospital, Fourth Military Medical University, Xi’an 710032, China; 2 Department of Radiology, Xijing Hospital, Fourth Military Medical University, Xi’an 710032, China; 3 Department of Anesthesiology, Shaanxi Provincial Hospital, Xi’an 710068, China; 4 Department of Anesthesiology, School of Medicine, Stony Brook University, New York 11794-8480, USA

## Abstract

Recent studies have demonstrated that adiponectin (APN) attenuates cerebral ischemic/reperfusion via globular adiponectin (gAD). However, the therapeutic role of gAD in cerebral ischemic injury in type 1 diabetes mellitus (T1DM) remains unclear. Our results showed that gAD improved neurological scores and reduced the infarct volumes in the 8-week T1DM (T1DM-8W) mice, but not in the 2-week T1DM (T1DM-2W) mice. Moreover, the ischemic penumbra APN levels increased and peaked in T1DM-2W mice, and reduced to normal in T1DM-8W mice, while the APN receptor 1 (AdipoR1) expression change was the opposite. Administration of rosiglitazone in T1DM-2W mice up-regulated the expression of AdipoR1 and restored the neuroprotection of gAD, while intracerebroventricular injection of AdipoR1 small interfering RNA (siRNA) in T1DM-8W mice reversed it. Furthermore, the expression of p-PERK, p-IRE1 and GRP78 were increased whereas the expressions of CHOP and cleaved caspase-12 as well as the number of apoptotic neurons were decreased after gAD treatment in T1DM-8W mice. These beneficial effects of gAD were reversed by pretreatment with AdipoR1 siRNA. These results demonstrated a dynamic dysfunction of APN/AdipoR1 accompanying T1DM progression. Interventions bolstering AdipoR1 expression during early stages and gAD supplementation during advanced stages may potentially reduce the cerebral ischemic injury in diabetic patients.

Diabetes mellitus (DM) is a complex metabolic disease and a major public health concern in China, with a prevalence rate of 9.7%^1^. Studies have found that diabetic patients have double the risk of suffering from stroke and 1-fold excess risk of stroke recurrence as compared to the normal population^2^. Moreover, the mortality and morbidity of stroke also increases in DM^3^. Therefore, the reduction of cerebral ischemic injury in DM is a major concern for scientists.

Adiponectin (APN) is regarded as an adipocyte-specific adipocytokine. Primary sequence analysis has revealed that full-length APN has four main domains, with the globular segment (gAD) at the carboxy terminus being much more potent than the full protein^4^. APN has multiple functions not only in the peripheral tissues, but also in the central nervous system. A previous study has confirmed that APN enhances tolerance against brain ischemia, with APN-KO mice suffering greater infarct volumes than wild-type mice^5^. Previous studies suggested the protective action of APN via an endothelial nitric oxide synthase-dependent mechanism, while Chen *et al.* confirmed the anti-inflammatory action of APN against cerebral ischemia/reperfusion (I/R) injury^6^. However, the contribution of other mechanisms to the neuroprotective action of APN requires further investigation.

Endoplasmic reticulum (ER) stress plays a critical role in various kinds of diseases including DM and neurodegeneration^7^. In the event of severe damage, apoptosis would be activated through ER stress-related unfolded protein response, leading to the elimination of damaged cells^8^. Accumulating evidences have suggested the involvement of ER stress in the regulation of post-cerebral I/R neuronal apoptosis^9^. Moreover, attenuation of ER stress could be a potential target for interfering brain I/R injury^10^. Recent data has suggested that the protective effect of gAD against myocardial I/R was modulated through suppressing of ER stress^11^. The involvement of ER stress in ischemia-induced vascular pathology in type II DM has also been proved^12^.

The current study aimed to investigate the neuroprotective effect of gAD on diabetic cerebral I/R model and further explore the underlying mechanisms. We assumed that with a proper therapeutic window, gAD could induce an anti-apoptotic effect against cerebral I/R injury through ER stress-related signaling pathway.

## Results

### gAD ameliorated cerebral I/R injury in T1DM-8W, but not in T1DM-2W mice

**Figure 1:**
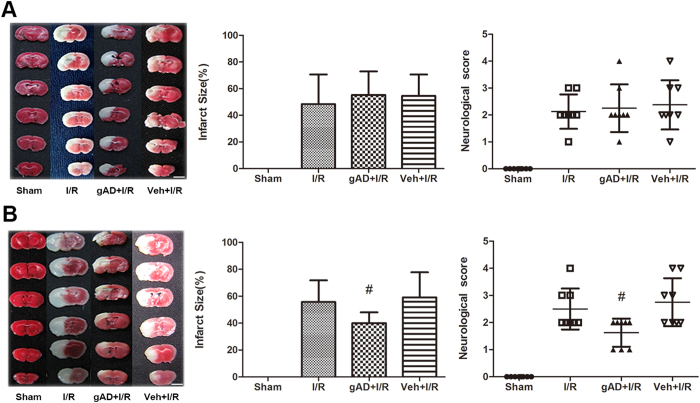
Varied effects of gAD against cerebral I/R. Neurological scores and representative samples of TTC-stained brain sections from **(A)** T1DM-2W and **(B)** T1DM-8W mice. Cerebral infarct size was assessed by TTC staining. TTC-stained areas (red) indicate viable tissue while the white area shows the ischemic region. gAD significantly improved the neurological scores and decreased the infarct volumes in 8 W DM mice but not in the 2 W DM mice. Data are expressed as mean ± SD (n = 8 per group). ^#^*P *< 0.05 vs. I/R group. Scale bars = 5 mm.

gAD failed to ameliorate I/R injury in T1DM-2W mice. There was no statistical difference in the neurological behavior among I/R, gAD + I/R, and Veh + I/R groups (*P *> 0.05), and the infarct size 24 h after reperfusion in all these groups was comparable (*P *> 0.05). Furthermore, the neurological behavior scores in these groups were significantly deteriorated as compared with the Sham group (*P* < 0.05, Fig. 1A). On the other hand, the gAD + I/R group demonstrated significant improvements in neurological deficit scores 24 h after I/R compared with the I/R group in T1DM-8W mice (*P *= 0.028). Mice receiving gAD from 8-week DM group showed a significant decrease in infarct size 24 h after I/R compared with the I/R group (*P *= 0.009). No significant difference was observed between I/R and Veh + I/R groups (*P *= 0.79, Fig. 1B).

### Adiponectin levels increased at 2-week and decreased at 8-week T1DM

**Figure 2:**
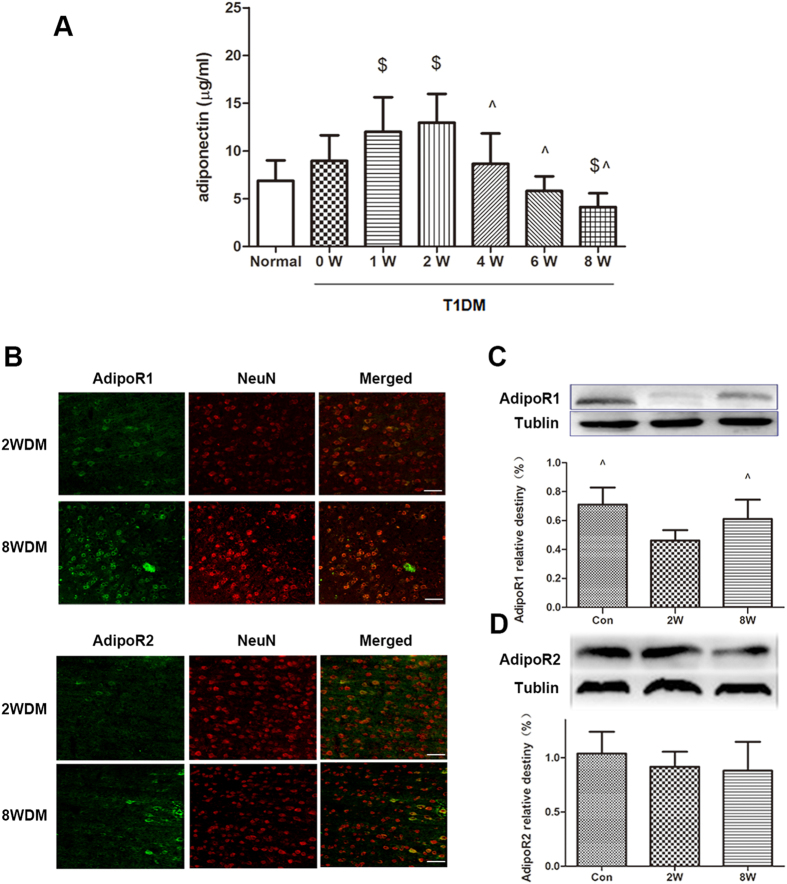
Dynamic alterations of APN and AdipoRs during T1DM progression. **(A)** Total APN levels were determined by ELISA after various T1DM durations. Data are expressed as mean ± SD (n = 8 per group). ^$^P < 0.05 vs. Control group; ^^^*P* < 0.05 vs. 2 W group. **(B)** Representative double immunofluorescence staining for AdipoR1, AdipoR2 (green), and neuron (NeuN, red) in brain sections. Scale bars = 20 μm. **(C,D)** Cerebral expression of AdipoR1 and AdipoR2 was determined by Western blot analysis. The upper part is the photograph of AdipoRs and their corresponding Tublin bands. Data are expressed as mean ± SD (n = 4 per group). ^^^*P *< 0.05 vs. 2 W group.

We assessed APN in cerebral tissues by ELISA at various time points during T1DM progression. As shown in Fig. 2A, cerebral APN levels markedly increased after 1-week T1DM duration and peaked at 2-week T1DM; the levels then gradually decreased through the remainder of the study, up to 8 weeks.

### Cerebral AdipoR1 was up-regulated in T1DM-8W mice, but not in T1DM-2W mice

A small number of AdipoR1-positive cells were observed in the brain tissue of the T1DM-2W mice. However, the number of AdipoR1-positive neurons significantly increased in the T1DM-8W group. There was no difference in the number of AdipoR2-positive cells between the two groups (Fig. 2B). The same results were observed via Western blot analysis with higher AdipoR1 expression (*P* = 0.001; Fig. 2C) in the T1DM-8W group, but not in the AdipoR2 group (*P* > 0.05, Fig. 2D).

### Up-regulation of AdipoR1 expression restored neuroprotection of gAD in T1DM-2W mice

**Figure 3:**
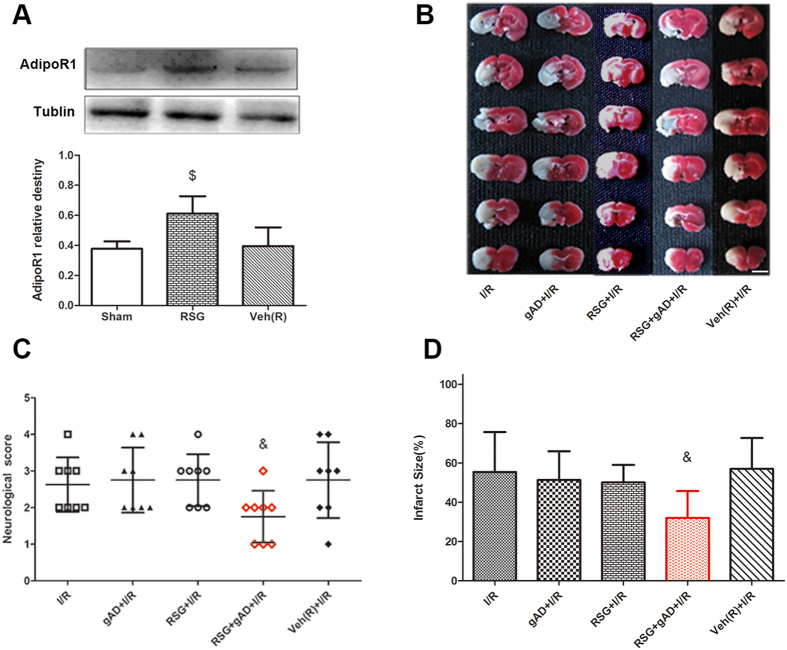
Effect of rosiglitazone on neuroprotection of gAD in T1DM-2W mice. **(A)** RSG significantly up-regulated the protein expression of AdipoR1 indicated by Western blot analysis (n = 4 per group). The upper part is the photograph of AdipoR1 and its corresponding Tublin bands. ^$^*P* < 0.05 vs. Sham group. **(B–D)** Pretreatment with RSG significantly improved the protective effect of gAD in 2-week DM mice, with lower neurological scores and decreased infarct volumes (n = 8 per group). ^&^*P* < 0.05 vs. gAD + I/R group. Scale bars = 5 mm.

According to the Western blot result, AdipoR1 was significantly increased in the RSG-treated group, while it showed no change in the Vehicle group (Fig. 3A).

The infarct volume in RSG + gAD + I/R group mice was significantly decreased as compared to that in gAD treated group 24 h after I/R in T1DM-2W mice (14.7% ± 4.3% vs. 31.2% ± 5.8%, *P *= 0.038, gAD + I/R vs. RSG + gAD + I/R). gAD failed to ameliorate the behavioral deficits while the neurological behavior outcome in mice pretreated with RSG was significantly improved 24 h after reperfusion injury (*P *= 0.028, gAD + I/R vs. RSG + gAD + I/R). There was no significant difference in the infarct volume or behavioral deficits score among I/R, gAD + I/R, and Veh (R) + I/R groups in T1DM-2W mice (Fig. 3B–D).

### Knockdown of AdipoR1 expression reversed neuroprotection of gAD in T1DM-8W mice

**Figure 4:**
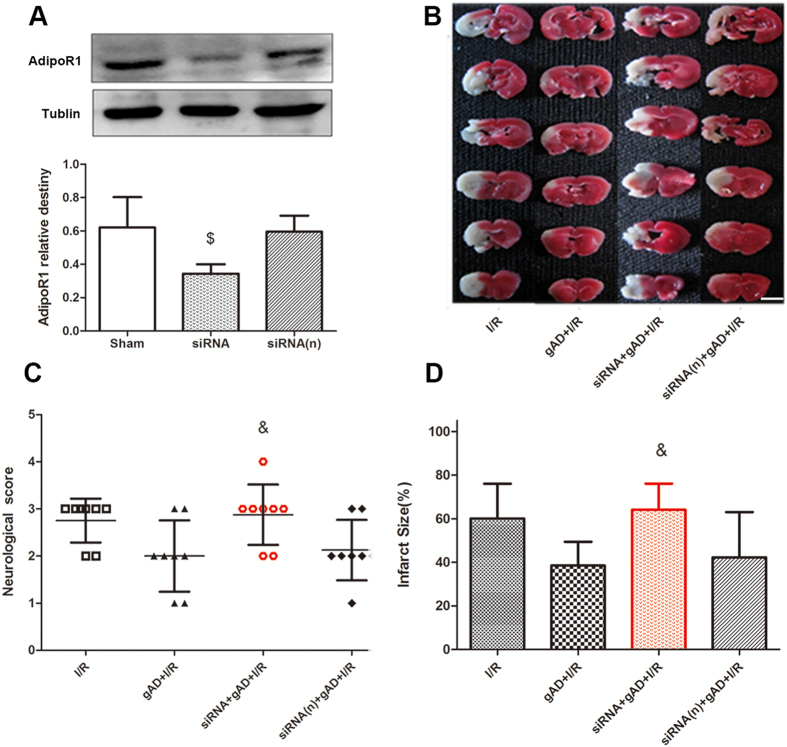
Effect of AdipoR1 siRNA on neuroprotection of gAD in T1DM-8W mice. **(A)** AdipoR1 siRNA significantly down-regulated the protein expression of AdipoR1 indicated by Western blot analysis (n = 4 per group). The upper part is the photograph of AdipoR1 and its corresponding Tublin bands. ^$^*P* < 0.05 vs. Sham group. **(B–D)** Pretreatment with AdipoR1 siRNA attenuated the protective effect of gAD in 8-week DM mice with disruptive neurological behavior and increased infarct volumes, whereas that with AdipoR1 negative siRNA showed no such effect. Values are presented as mean ± SD (n = 8 per group). ^&^*P* < 0.05 vs. gAD + I/R group. Scale bars = 5 mm.

After intracerebroventricular administration of AdipoR1 siRNA or control siRNA, the AdipoR1 protein in DM mice brains was analyzed by Western blot. The results indicated that AdipoR1 expression was down-regulated by AdipoR1 siRNA compared to Sham or siRNA (n) (Fig. 4A).

Compared with the gAD + I/R group, the infarct volume in mice pretreated with AdipoR1 siRNA was significantly increased 24 h after I/R in T1DM-8W mice (14.7% ± 4.3% vs. 31.2% ± 5.8%, *P *= 0.038, gAD + I/R vs. siRNA + gAD + I/R). gAD could ameliorate the behavioral deficits while the neurological behavior outcome in siRNA + gAD + I/R group had significantly worsened 24 h after cerebral I/R (*P *= 0.028, gAD + I/R vs. siRNA + gAD + I/R). No significant difference in infarct volume or neurological scores was observed between the gAD + I/R and siRNA(n) + gAD + I/R groups in T1DM-8W mice (Fig. 4B–D).

### gAD alleviated ER stress in T1DM-8W mice with cerebral I/R injury

**Figure 5:**
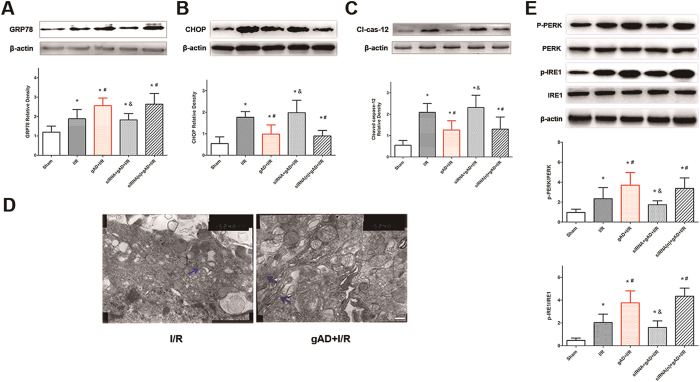
Expression of ER stress protein and ER morphology in T1DM-8W mice . Western blot analyses were performed for **(A)** GRP78, **(B)** CHOP, and **(C)** cleaved caspase-12 **(E)** PERP, p-PERP, IRE-1, p-IRE-1 in the penumbra of different groups. The upper part is the photograph of ER stress proteins and their corresponding β-Actin bands. After MCAO, all the ER stress-related proteins were up-regulated. After gAD treatment, the expression of p-PERP, p-IRE-1, GRP78 was increased while that of CHOP and cleaved caspase-12 was decreased. The effects of gAD were reversed by pretreatment with AdipoR1 siRNA. Values are presented as mean ± SD (n = 4 per group). ^*^P < 0.05 vs. Sham group. ^#^*P* < 0.05 vs. I/R group. ^&^*P* < 0.05 vs. gAD + I/R group. **(D)** Representative electron microscopy images of ER morphology. After I/R injury, the ER was disaggregated and difficult to identify. With gAD treatment, some ER could be identified as having regular shape. Scale bars = 200 nm.

GRP78, CHOP, and caspase-12 are markers of ER stress while PERK and IRE1 are 2 major ER stress sensor protein. In the present study, the expressions of these proteins from the penumbra were investigated in T1DM-8Wmice. At 24 h after reperfusion, the expression of p-PERK, p-IRE1 and ER stress chaperone protein GRP78 significantly increased in gAD + I/R group (*P *< 0.05, gAD + I/R vs. I/R). However, AdipoR1-specific siRNA reversed this effect of gAD as indicated by the p-PERK, p-IRE1 and GRP78 expression significantly reduced in siRNA + gAD + I/R group (Fig. 5A,E).

The results revealed that I/R markedly increased expressions of CHOP (*P* = 0.001; I/R vs. Sham) and activated caspase-12 (*P *= 0.001, I/R vs. Sham), while gAD lowered them (*P *< 0.05; I/R vs. gAD + I/R, Fig. 5B [CHOP] and *P *= 0.028; I/R vs. gAD + I/R, Fig. 5C [activated caspase-12]). However, when pretreated with AdipoR1 siRNA, the expressions of CHOP (*P *< 0.05; gAD + I/R vs. siRNA + gAD + I/R) and activated caspase-12 protein were significantly lower than those in gAD group (*P *< 0.05; gAD + I/R vs. siRNA + gAD + I/R).

The ultrastructure of neurons was examined by TEM. The ER morphology changes such as clumping, margination, and condensation were considered the most important evidence of injury. In I/R group, the damaged ER was condensed, verifying degeneration, while gAD treatment could ameliorate the injury of ER (Fig. 5D).

### gAD inhibited neuronal apoptosis in T1DM-8W mice with cerebral I/R injury

**Figure 6:**
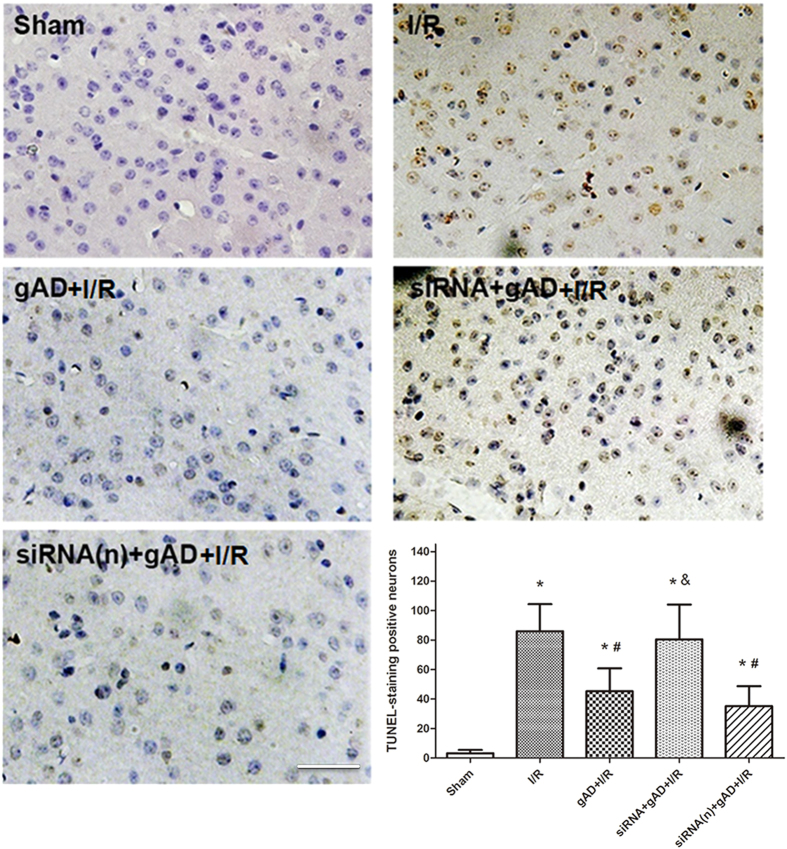
Neuronal apoptosis in T1DM-8W mice with cerebral I/R. Representative photomicrographs of TUNEL in the ischemic penumbra by immunohistology and quantitative analysis. gAD significantly decreased the number of TUNEL-positive cells. AdipoR1 siRNA reversed the anti-apoptotic effect of ER, whereas control siRNA showed no such effect. Scale Bar = 20 μm. Values are presented as mean ± SD (n = 5 per group). ^*^P < 0.05 vs. Sham group. ^#^*P* < 0.05 vs. I/R group. ^&^*P* < 0.05 vs. gAD + I/R group.

Figure 6 showed the representative photomicrographs of TUNEL staining in the ischemic penumbra from different groups. In the Sham group, few TUNEL-positive cells were observed in the brain tissue 24 h after surgery. The number of TUNEL-positive neurons significantly increased in I/R group (*P < *0.001, I/R vs. Sham) and markedly diminished in the gAD group (*P *= 0.003, gAD + I/R vs. I/R). However, when pretreated with AdipoR1 siRNA rather than the control siRNA, more apoptotic neurons were detected compared with gAD group (*P *= 0.038, gAD + I/R vs. siRNA + gAD + I/R).

## Discussion

DM is a severe metabolic disorder with a worldwide prevalence of 6.4%. The total diabetic population is expected to reach 438 million^13^. APN is a cytokine that is primarily synthesized and secreted by fat cells. It has been proven to play a critical role in DM^14^,^15^. Stroke is a severe and common complication of DM. Diabetic patients are at an increased risk for cerebral ischemic injury^16^. Although there still remain some controversies and arguments^17^,^18^, some of the previous studies proved that low APN level is correlated with the severity of stroke and negatively related with brain infarct volume, as well as with the NIHSS score^19^,^20^. It is also associated with atherosclerosis^21^. A study by Sasaki *et al.* showed that circulating APN level is temporarily decreased in acute ischemic stroke patients, and then recovers to baseline levels 14 days later^22^. It has also been proved that APN is effective against cerebral I/R through anti-inflammatory and anti-oxidative mechanisms^23^,^24^. Moreover, APN supplementation reduced the myocardial ischemic injury in T1DM mice^25^. Increased plasma APN was associated with decreased risk of Type 2 DM and thus, reduced risk of cardiovascular disease^26^. As globular APN is the main functional fragment of APN, our present study proved that gAD has a neuroprotecitve effect against cerebral I/R injury in 8-week DM mice. However, the protection was not observed in the 2-week DM mice.

To evaluate the dynamic therapeutic effects of gAD against cerebral ischemic injury in diabetic models, we focused on the change in APN level during the course of DM. We observed that APN levels peaked in the second week of T1DM and decreased during DM progression. Some studies indicated that APN concentration is positively related to cardiovascular risk in T1DM patients^27^,^28^, while others showed the opposite^29^,^30^. The dynamic change in the APN level might at least partially explain the previous contradictory results of APN in T1DM.

APN exerts its biological effects via its two receptors, AdipoR1 and AdipoR2, and previous studies offered some evidences for the expression of AdipoRs in the central nervous system^31^. However, the alteration of cerebral AdipoRs expression in a diabetic model, which would be essential for understanding the APN-AdipoR signaling pathway in the metabolic progression, has not been studied earlier. From our result, AdipoR1 expression decreased 2 weeks after the onset of DM but increased in the later stage, i.e. 8 weeks. It is interesting to note that no significant difference in AdipoR2 was observed in either phase. These results might help deduce the different roles of the 2 receptors in the course of DM.

On one hand, we tried to explain the possible reason for the failure of gAD in 2-week DM mice. Increased up-regulation of endogenous APN was observed at the early stage; however, it failed to protect against cerebral I/R. The administration of gAD in 2-week DM mice also showed no significant differences with respect to the infarct size and neurological behavior. This might be related to the lower expression of AdipoR1. To further prove our hypothesis, we used RSG as a peroxisome proliferator activated receptor gamma activator. It could increase AdipoR1 expression, thus activating the downstream AMPK-related signaling pathway^32^. After RSG injection, a neuroprotective effect of gAD was restored in 2-week T1DM mice, evidenced by a decreased infarct volume and improved neurological behavior. Therefore, we proved that failure of gAD in the early stage of DM was at least partially associated with decreased AdipoR1 expression.

On the other hand, to explain the mechanism of gAD in 8-week DM mice, we proved that AdipoR1 siRNA reversed the protective effect of gAD. This indicated the important role of AdipoR1 in the neuroprotective effect of gAD. Furthermore, the downstream signaling pathway was also explored. Cerebral ischemic reperfusion injury, together with DM, triggers multiple overlapping signaling pathways in the brain. It has been suggested that ER stress is involved in cerebral ischemic pathophysiology and neuronal apoptosis^33^. It also contributes to autoimmunity and insulin resistance in diabetic patients^34^.

ER chaperones are highly sensitive to I/R injury and are representative of ER stress^35^. We found that damaged ER morphology was ameliorated with gAD. ER stress markers such as GRP78, CHOP, and caspase-12 were enhanced after I/R injury and reversed by gAD treatment in T1DM-8W mice (except for GRP78, which was further increased). GRP78 regulates protein folding and facilitates protein translocation in ER^36^. A previous study indicated that up-regulation of GRP78 attenuated the induction of CHOP and reduced ER stress-related apoptosis^37^. Therefore, the increased expression of GRP78 by gAD might contribute to the neuroprotective effect against I/R injury. Moreover, CHOP as a proapoptotic transcription factor plays a key role in ER stress-related apoptosis, while caspase-12 as a murine protein dissociates from the ER membrane and gets activated after ER stress^38^,^39^. Our results showed that the activation of both CHOP and caspase-12 was significantly suppressed by gAD. In addition, with the knockdown of AdipoR1, the regulation of gAD on GRP78, CHOP, and caspase-12 expression were reversed, which showed the anti-ER stress was in close relationship with the gAD-AdipoR1 signaling pathway. For further exploring the possible molecular mechanisms of gAD, ER stress sensor proteins R-like endoplasmic reticulum kinase (PERK) and inositol-requiring enzyme 1 (IRE1) in neurons were examined. PERK and IRE1 are major ER stress sensor proteins. ER stress leads to isolation of GRP78 from PERK and IRE1, which in turn activates downstream ER stress response. Previous study suggested that PERK and IRE1 phosphorylation are crucial for maintaining the balance between cell survival and death^40^,^41^. Our results indicated that gAD could lead to the further activation of PERK and IRE1 pathways, which might lead subsequently to down-regulation of the pro-apoptotic protein CHOP and caspase12, while blocking AdipoR1 could reverse the effect of gAD. The alterations in p-IRE1 and p-PERK levels were consistent with those in GRP78 level. Dong *et al.*, reported that ER stress proteins were unaffected by adiponectin in db/db diabetic obese mice^42^. The differences between results might due to the different type of mice as well as different injury model. Moreover, neuronal apoptosis is in close relationship with ER stress. Dysfunction of this organelle occurring at an early stage after ischemia might be the initial step of apoptotic cascades in neurons^43^. This apoptosis could reflect the function of the ER to some extent, while prolonged ER stress would ultimately lead to cell apoptosis. Therefore, the anti-apoptotic effect of gAD and the role of AdipoR1 in our study further support our assumption.

This study also had some limitations. Firstly, although we offered an evidence for the dynamic dysfunction of APN/AdipoR1 during T1DM progression as well as the protective role of gAD, the mechanisms might not be applicable for explaining type 2 diabetic models with highly variable metabolic properties. Further studies are required to determine the role of gAD and AdipoRs during type 2 DM and in different models. Secondly, our previous study demonstrated the protective effect of gAD against ischemic stroke in normal adult mice^24^. In the current study we focused only on T1DM and did not compare the differences between normal and DM mice. Thirdly, the study is not mechanistic enough. Our previous study showed that the neuroprotective action of gAD against ischemic stroke in normal adult mice resulted from the promotion of antioxidant capacity by inhibiting the NOX2 signaling system. Moreover, we also proved that electroacupuncture alleviated cerebral ischemic injury in diabetic mice stroke model through adiponectin/AdipoR1-mediated signaling pathway, in which glycogen synthase kinase 3-β might be one crucial molecule in the downstream pathway of AdipoR1^44^. Nevertheless, the possible molecular mechanism of gAD for treating diabetic stroke should be addressed in future researches.

Above all, our study indicated the therapeutic window of globular APN against cerebral ischemic injury in diabetic mice. Accordingly, the dynamic dysfunction of APN and AdipoR1 could partially explain the deficient neuroprotective effect of gAD against I/R in 2-week T1DM mice but efficiently for 8-week T1DM mice. Moreover, ER stress-apoptosis signal pathway might play an important role for I/R injury in the late stage of T1DM mice, and gAD-AdipoR1 could mitigate the injury. Interventions bolstering AdipoR1 expression during early stages and gAD supplementation during advanced stages may potentially reduce the cerebral ischemic injury in diabetic patients. We hope that our current results provide a reliable theoretical basis for future clinical treatment of ischemic stroke patients with DM.

## Materials and Methods

### Experimental protocol

All experiments were performed in accordance with the National Institutes of Health Guide for the Care and Use of Laboratory Animals (NIH Publications No. 80-23) revised in 1996, and associated guidelines. The experimental protocol used in this study was approved by the Ethics Committee for Animal Experimentation of the Fourth Military Medical University, Xi’an, China, and the study was conducted according to the Guidelines for Animal Experimentation of the same university. Adult male C57BL/6 mice weighing 20–25 g were purchased from the Experimental Animal Center of the University and housed under controlled conditions with a 12-h light/dark cycle, at a temperature of 21 ± 2 °C and a humidity of 60–70% for at least one week prior to experimentation. The animals were allowed free access to standard rodent diet and water.

T1DM mice were induced via injection of low dose of streptozotocin (50 mg/kg, ip) for five consecutive days. The blood sugar was detected 10 days after the first day of injection, and the mice with blood glucose level >13.3 mmol/L were considered as successful models for the experiment. This time point was defined as T1DM Day 0. The blood glucose and weight of the mice were measured weekly thereafter.

#### Experiment I

In order to test the neuroprotective effect of gAD against cerebral I/R injury in T1DM mice, 64 male mice were randomly assigned to Sham, I/R, gAD + I/R (gAD treatment, i.c.v. 30 min before I/R), or Veh + I/R (Vehicle as phosphate buffered saline [PBS] i.c.v. 30 min before I/R) treatment groups in 2-week T1DM (T1DM-2W) mice and 8-week T1DM (T1DM-8W) mice. I/R was performed to induce focal cerebral ischemic stroke.

#### Experiment II

In order to assess the dynamic change in APN level and alteration of AdipoR expression in diabetic mice, 88 mice were involved and the group was divided as Control, T1DM-2W mice, and T1DM-8W mice.

#### Experiment III

In order to evaluate the effect of AdipoR1 on gAD-induced neuroprotection in T1DM-2W mice, 40 animals were randomly divided into I/R, gAD + I/R, RGS + I/R ([rosiglitazone, RGS], intraperitoneal injection, 2 mg/kg, 4 h before I/R), RSG + gAD + I/R, and Veh (R) + I/R groups. RGS is an anti-diabetic drug, and acts as a peroxisome proliferator-activated receptor gamma activator. In the current study, it was used to up-regulate AdipoR1 expression. Its doses were based on the previously published results and those of our preliminary study^4^,^6^,^45^,^46^.

#### Experiment IV

In order to investigate the role of AdipoR1 in T1DM-8W mice and to further explore the possible mechanisms of gAD, 84 T1DM-8W mice were assigned to I/R, gAD + I/R, siRNA + gAD + I/R (AdipoR1-specific siRNA, 0.3 nmol/μL, 1.2 nmol, i.c.v, 3 days before I/R), and siRNA (n) + gAD + I/R (negative siRNA, 3 nmol/μL, 1.2 nmol, i.c.v, 3 days before I/R) groups.

### Drug injection

For gAD pretreatment, the mice were deeply anesthetized with 2.5% isoflurane and placed on a stereotaxic frame (Stoelting, Wood Dale, IL, USA). Recombinant mouse globular APN (gAD, produced in Escherichia coli [Peprotech EC, London, UK]) was injected into cerebroventricularl (i.c.v, bregma: 1.0 mm lateral, 0.5 mm posterior, 2.0 mm deep) at 30 min before ischemia and in a dose of 0.1μg/g body weight (diluted in PBS) as previously published^45^. PBS (pyrogen-free, 0.01 M, pH 6.8-7.4, 2 μL) was also injected as a vehicle control.

### Transient focal cerebral ischemia

Focal cerebral ischemia was induced by middle cerebral artery occlusion (MCAO, under anesthesia by inhaling 2.5% isoflurane) in mice using the intraluminal filament technique, as previously described^5^. The suture was withdrawn 90 min after MCAO. Regional cerebral blood flow was measured by laser Doppler flowmetry (Periflux 5000, Perimed, Stockholm, Sweden) with a flexible probe fixed on the skull, 1 mm posteriorly and 6 mm laterally from the bregma and monitored throughout surgery.

### Neurological score evaluation and infarct assessment

At 24 h after reperfusion, neurological deficit scores were assessed in a blinded manner according to the the 5-point scale method^47^. Briefly, normal motor function was scored as 0, flexion of the contralateral torso and forelimb on lifting the animal by the tail as 1, circling to the contralateral side but normal posture at rest as 2, leaning to the contralateral side at rest as 3, and no spontaneous motor activity as 4. After neurological score evaluation, the animals were decapitated under deep anesthesia (pentobarbital, 150 mg/kg body weight), and their brains were removed, frozen, and sectioned (1 mm thick). The brain sections were stained with 2% (w/v) 2,3,5-Triphenyltetrazolium chloride (TTC; Sigma, St. Louis, MO, USA) to evaluate infarct volume, as previously described^48^.

### ELISA

Samples of the ischemic penumbra were homogenized in cold RIPA lysis buffer with 1× Roche complete protease inhibitor cocktail and 1 mM phenylmethylsulfonyl fluoride on ice. The tissue extract was centrifuged at 12,000 × g at 4 °C for 30 min and the supernatant was removed and stored at −80 °C until use. The total APN concentration was measured by ELISA according to the instructions (Westang Biotechnology, Shanghai, China).

### Immunofluorescence staining

For analysis of AdipoR1 and AdipoR2 expression, the mice from Experiment II were anesthetized and perfused with PBS and 4% paraformaldehyde 24 h after reperfusion. The brains were cut, prepared, and stained as described in our previous study^49^. Different brain areas in each section from the same part of the brain in each group were observed and photographed by a microscope (20×; Olympus, BX51, Japan).

### Western Blot Analysis

Immunoblot analysis was performed according to a previously described method^48^. The following primary antibodies were used: anti-phospho-IRE, anti-IRE1 (1:1000; Cell Signaling Technology, Danvers, MA, USA), anti-phospho-PERK, anti-PERK (1:1000; Santa Cruz Biotechnology, Santa Cruz, CA, USA), anti-GRP 78 antibody (1:200; Abcam, Cambridge, MA, USA), anti-CHOP antibody (1:1000; Abcam, Cambridge, MA, USA), anti-cleaved caspase-12 (1:200; Abcam, Cambridge, MA, USA), anti-AdipoR1 antibody (1:1000; BD Bioscience, Franklin Lakes, NJ, USA), anti-AdipoR2 antibody (1:1000; BD Bioscience, Franklin Lakes, NJ, USA), anti-β-actin antibody (1:10000; Santa Cruz Biotechnology, Santa Cruz, CA, USA), and anti-β-tubulin antibody (1:10000; Santa Cruz Biotechnology, Santa Cruz, CA, USA). The membranes were then washed and incubated with suitable secondary antibodies for 1 h at room temperature. Antigens were detected using the chemiluminescence technique (Amersham Pharmacia Biotech Piscataway, NJ, USA). Image analysis was completed using the Bio-Rad Laboratories (Hercules) analysis software.

### Transmission electron microscopy

Vibratome sections from the penumbra were selected and transferred onto the phosphate buffer. The tissues were rinsed in buffer and postfixed with 1% osmium tetroxide for 1 h, subjected to a graded ethanol dehydration series, and infiltrated using a mixture of one-half propylene oxide and resin overnight. After 24 h, the tissues were embedded in resin. The 120-nm sections were cut and stained with 4% uranyl acetate for 20 min and 0.5% lead citrate for 5 min. The ultrastructure of the hippocampus was observed under transmission electron microscopy (TEM [Philips Tecnai 10, Philips, Holland]).

### TUNEL staining

Neuronal cell apoptosis in the ischemic penumbra was quantified using a commercially available terminal deoxynucleotidyl transferase dUTP nick-end labeling (TUNEL) staining kit (Roche Diagnostics, Mannheim, Germany) as previously described^50^. The total number of TUNEL-positive cells in the right hemisphere were counted in three different fields for each section in a naive manner using light microscopy (BX51; Olympus, Tokyo, Japan), and data from four animals in each group were averaged.

### Statistical analysis

All values, excluding neurological scores, were expressed as mean ± SD. Statistical significance was tested by one-way analysis of variance, and differences between the groups were detected using the Dunnett’s test. The neurological scores, presented as a median, were analyzed by a nonparametric method (Kruskal-Wallis test) followed by the Mann-Whitney U test with Bonferroni correction. *P *< 0.05 was considered statistically significant.

## Additional Information

**How to cite this article**: Song, W. *et al.* Therapeutic window of globular adiponectin against cerebral ischemia in diabetic mice: the role of dynamic alteration of adiponectin/adiponectin receptor expression. *Sci. Rep.* **5**, 17310; doi: 10.1038/srep17310 (2015).
